# Learn 2 Move 16-24: effectiveness of an intervention to stimulate physical activity and improve physical fitness of adolescents and young adults with spastic cerebral palsy; a randomized controlled trial

**DOI:** 10.1186/1471-2431-10-79

**Published:** 2010-11-05

**Authors:** Jorrit Slaman, Marij E Roebroeck, Jetty van Meeteren, Wilma M van der Slot, Heleen A Reinders- Messelink, Eline Lindeman, Henk J Stam, Rita J van den Berg-Emons

**Affiliations:** 1 Department of Rehabilitation Medicine and Physical Therapy, Erasmus MC, University Medical Center, Rotterdam, The Netherlands; 2 Rijndam Rehabilitation Center, Rotterdam, The Netherlands; 3 Rehabilitation Center 'Revalidatie Friesland', Beetsterzwaag, The Netherlands; 4 Department of Rehabilitation Medicine, University Medical Center Groningen, Groningen, The Netherlands; 5 Department of Research and Development, Rehabilitation Center De Hoogstraat, Utrecht, The Netherlands; 6 Department of Rehabilitation, Nursing Science and Sports, University Medical Center, and Rudolf Magnus Institute of Neuroscience, Utrecht, The Netherlands

## Abstract

**Background:**

Persons with cerebral palsy (CP) are at risk for developing an inactive lifestyle and often have poor fitness levels, which may lead to secondary health complications and diminished participation and quality of life. However, persons with CP also tend not to receive structural treatment to improve physical activity and fitness in adolescence, which is precisely the period when adult physical activity patterns are established.

**Methods:**

We aim to include 60 adolescents and young adults (16-24 years) with spastic CP. Participants will be randomly assigned to an intervention group or a control group (no treatment; current policy). The intervention will last 6 months and consist of three parts; 1) counselling on daily physical activity; 2) physical fitness training; and 3) sports advice. To evaluate the effectiveness of the intervention, all participants will be measured before, during, directly after, and at 6 months following the intervention period. Primary outcome measures will be: 1) physical activity level, which will be measured objectively with an accelerometry-based activity monitor during 72 h and subjectively with the Physical Activity Scale for Individuals with Physical Disabilities; 2) aerobic fitness, which will be measured with a maximal ramp test on a bicycle or armcrank ergometer and a 6-minute walking or wheelchair test; 3) neuromuscular fitness, which will be measured with handheld dynamometry; and 4 body composition, which will be determined by measuring body mass, height, waist circumference, fat mass and lipid profile.

**Conclusions:**

This paper outlines the design, methodology and intervention of a multicenter randomized controlled trial (LEARN 2 MOVE 16-24) aimed at examining the effectiveness of an intervention that is intended to permanently increase physical activity levels and improve fitness levels of adolescents and young adults with CP by achieving a behavioral change toward a more active lifestyle.

**Trial registration:**

Dutch Trial Register; NTR1785

## Background

Cerebral palsy (CP) occurs in 1.5 to 3.0 of 1000 live births and is the most common cause of physical disability in pediatric rehabilitation medicine [1]. Representing a group of permanent disorders of the development of movement and posture, it causes activity limitation that is attributed to non progressive disturbances that occurred in the developing fetal or infant brain [2] which can lead to diminished quality of life, participation and health in adulthood [3-5].

Participation in regular physical activity (PA) provides psychological and physiological benefits in adolescents [6]. In addition, it reduces both the deterioration of mobility-related activities [7] and the development of secondary health problems later in life [8]. However, performing physical activities is often burdensome in adolescents and adults with CP due to reduced muscle mass and inefficient locomotion [9]. This might, together with the distinctly subnormal fitness levels of persons with CP [10], explain why they are at risk of developing an inactive lifestyle. Such an inactive lifestyle has been found in several studies of bilateral spastic CP populations [11-13], but it contrasts with the more active lifestyle of individuals with unilateral spastic CP [14].

Persons develop their adult PA lifestyle in adolescence [15,16]. Therefore, encouraging an active lifestyle and improving physical fitness at this age seems important. Nevertheless, in adolescence and young adulthood, persons with CP tend not to receive structural treatment to improve PA and fitness [17]. A recent review shows that children and adolescents with CP may benefit from exercise programs to improve physical fitness [18]. However, higher fitness levels do not automatically lead to a more active lifestyle [19,20]. Some evidence exists for the effectiveness of interventions aimed at stimulating an active lifestyle through individual counselling in adult populations with physical disabilities in both the short term [21] and long term [22]. It can be speculated that an intervention which combines individual counselling on daily PA with fitness training is ideal to increase PA and improve physical fitness in persons with CP. However, the effectiveness of such an intervention has, to our knowledge, never been evaluated in an adolescent or young adult population with CP.

The LEARN 2 MOVE 16-24 research project aims to evaluate the effectiveness and underlying working mechanisms on the short and long term of an Active Lifestyle and Sports Participation (ALSP) intervention. It is hypothesized that persons following the ALSP intervention will experience increased PA and improved physical fitness in both the short term and the long term (maintenance of effects) as the primary goal of the intervention is to achieve a behavioral change toward a more active lifestyle. The present paper describes the research design, methodology and intervention of the LEARN 2 MOVE 16-24 research project [23-25].

## Methods/design

### Ethical approval

Multicenter approval was granted by the Erasmus MC Medical Ethics Committee, The Netherlands. Local approval was granted by all participating centers.

### Study design

The study has a multicenter randomized controlled design. The experimental group will receive the ALSP intervention. The control group will receive no intervention to improve PA and fitness, which is current policy. Erasmus MC (Rotterdam), Rijndam Rehabilitation Center (Rotterdam), VU Medical Center (Amsterdam), Rehabilitation Center Amsterdam (Amsterdam), Rehabilitation Center De Hoogstraat (Utrecht) and Sophia Rehabilitation (Den Haag/Delft) will participate in this study.

### Blinding

This study has a single-blind research design; all measurements will be performed by assessors who are blind for group allocation and who have no involvement in the recruitment, randomization procedure or intervention.

### Setting

The ALSP intervention and the research measurements will be performed at each participant's house and at two university medical centers and four rehabilitation centers from which all participants will be recruited.

### Sample size

At least 50 participants are required to detect a difference of 30 minutes a day in PA level between the control and experimental group, with a power of 0.8 and an alpha of 0.05. To allow for dropouts, we aim to recruit at least 60 participants. This calculation is based on data from previous research conducted by our research group [13,14].

### Participants

#### Inclusion criteria

Adolescents and young adults with spastic unilateral or bilateral CP are eligible for inclusion if they meet each of the following criteria

• Age 16 - 24 years

• Gross Motor Functioning Classification System (GMFCS [26]) level I-IV

#### Exclusion criteria

Individuals will be excluded if they meet any of the following criteria

• Disabilities other than CP that affect PA or fitness level

• Contraindication for (maximal) exercise

• Severe cognitive disorders and insufficient comprehension of the Dutch language that preclude understanding the purpose of the project and its testing methods

• PA level at baseline exceeds the mean PA level + 2 SD of a CP population

### Recruitment

Each participating center will compile a list of its patients aged 16 - 24 years and diagnosed with CP. All patients will be checked on inclusion and exclusion criteria by their rehabilitation physician. An information letter and invitation to participate was sent to eligible participants. A second letter was sent 3 weeks later to non-responders.

### Randomization

After baseline measurement, participants will be stratified according to their GMFCS level to obtain an equal distribution of gross motor functioning between the two groups. Within each stratum and for each participating center, participants will be randomly allocated (1:1) to the experimental or control group.

### Intervention: Active Lifestyle and Sports Participation

The ALSP intervention lasts 6 months and was developed for adolescents and young adults with physical disabilities [27]. It aims to permanently increase PA and fitness levels by promoting behavioural changes toward a more active lifestyle (EMGO Institute VUmc, Amsterdam, The Netherlands; Health Partners & Io Solutions, Minneapolis, Minnesota, USA) and consists of three parts:

1. Counselling on daily PA

2. Physical fitness training

3. Sports advice

#### 1. Counselling on daily PA

Counselling on daily PA is the main component of the intervention and consists of six individual counselling sessions over a period of 6 months with a PA counsellor (e.g. physical therapist, movement therapist) who serves as a 'personal coach'. During these counselling sessions, participants will receive individual PA advice which primarily focuses on PA in daily life, and not necessarily on sports. The counselling sessions on daily PA are based on Motivational Interviewing (MI) which is defined as a directive, client-centered counselling style for eliciting behavioral changes by helping clients to explore and resolve ambivalence [28]. Techniques that will be applied in the ALSP intervention to invoke the spirit of MI are reflective listening, rolling with resistance, asking open-ended questions, summarizing during a conversation, and showing empathy for the participant. The stages of behavioral change of the Transtheoretical model [29] describe the stages a person experiences when changing his or her health behavior. This model structures the clinical role of MI during the counselling sessions [30]. The first counselling session will last 45 minutes. The five subsequent 20- to 30- minute sessions will be planned monthly. All counselling sessions for a participant will be supervised by the same counsellor. Each participating center will have its own counsellor and all involved counsellors will undertake two days of training to learn the basic principles of MI. The counsellors will also participate in a 1-day follow-up workshop to discuss their initial experiences and refresh their MI knowledge and skills. The first and last counselling session will be sound-recorded for latter analysis to ensure that MI had been applied correctly.

#### 2. Physical fitness training

Physical fitness training will consist of 12 weekly supervised sessions and 12 home sessions. These training sessions of approximately one hour consist of aerobic endurance, aerobic interval and strength training. Over these 12 weeks, aerobic exercise duration and aerobic intensity (as determined by the Karvonen formula [31]) are gradually increased. The peak heart rate (HR), which is required for the Karvonen formula, will be determined during a maximal ramp protocol. The required resting HR will be determined after 5 minutes of rest in a sitting position. During training, HR will be recorded using a Garmin Forerunner 60 heart rate monitor (Garmin Ltd., Olathe, KS, USA). The exercise program starts at a HR of 40% of the heart rate reserve (HRR) on a treadmill, cycle or armcrank ergometer. This will increase to a HR of 80% of the HRR at week 12 [32]. Also, strength exercises of the large muscle groups are included. The training load will be based on a percentage of the baseline 1 repetition maximum (1RM), which is the maximum load that a person can lift once. The training load will begin with one set of 10 to 15 repetitions at 30% of 1RM during week 1 and will increase toward three sets of 15 to 20 repetitions at 75% of 1RM during week 12 [32]. Slight adaptations to these guidelines will be allowed to ensure that the training is both feasible and challenging for each participant. Fitness equipment is available for upper extremities, lower extremities and abdominal muscles. For the training at home, participants will be instructed to perform aerobic exercises (walking/running, cycling, hand-cycling, wheelchair-driving, or swimming) and strength exercises (using Thera-Bands; Hygenic Corporation, Akron, OH, USA) of similar duration and intensity as the supervised training session. HR will also be recorded during home training sessions to evaluate whether participants accomplish the exercise instructions in terms of duration and intensity. After 12 weeks of supervised fitness training the possibilities will be explored to continue the fitness training in the day-to-day environment of each individual.

#### 3. Sports Advice

Sports advice includes sports counselling and sport-specific training. Sports counselling is based on the Rehabilitation & Sports Program [21,22]. During 2 to 4 sessions of approximately 30 minutes, sports history, preferences, possibilities, barriers and facilitators will be identified. This will result in an individualized sports advice, including information on available, accessible and appropriate sports facilities in the person's day-to-day environment. Aspects as transportation, finances, and supervision are also taken into account to ensure long-term benefits. Sport-specific training includes practicing the required skills to perform the sport and ensuring practice opportunities to match sports to each participant's interests and abilities. Examples of the sports to be included in the sport specific training are swimming, soccer, rowing, basketball, climbing, golf, hockey and racket sports. Participation in the sport-specific training sessions will depend on the interests and physical abilities of the participant.

## Measurements

### International Classification of Functioning, Disability and Health

**Figure 1:**
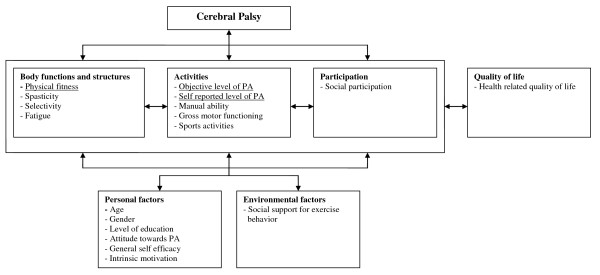
**ICF model with the outcome measures that will be assessed in this study**. Primary outcome measures are underlined.

The International Classification of Functioning, Disability and Health (ICF) model provides a unified language and framework for the description of health and health-related states [33]. This model identifies how a health condition, such as CP, can interact with features of the person and the environment to produce three levels of potential disablement: 1) impairments which occur at the level of body structures and functions; 2) activity limitations which occur at the level of performance of tasks or actions by the person; and 3) participation restrictions which occur at the level of participants in their social context. An overview of the ICF model with the parameters to be measured in the present study is shown in Figure 1. The primary outcome measures of this study are the objectively measured PA level, self-reported PA level and physical fitness. All outcome measures will be assessed: at baseline (T0); immediately after completion of the total intervention (6 months, T6) to establish the effectiveness of the ALSP intervention; and at 6 months following the intervention period (12 months, T12) to determine long-term effects. A limited set of outcome measures (physical fitness, health-related quality of life and fatigue) will be assessed after 3 months (T3) to establish the effectiveness of the fitness training of the ALSP intervention. A time schedule of the ALSP intervention and measurements is presented in Table 1. Comparable research equipment will be used in all participating centers.

**Table 1:** Time schedule of the Active Lifestyle and Sports Participation intervention.

Week	Measurements	Counselling on daily PA	Fitness training Supervised	Home-based	Sports advice
Week 1	Pre-test (T0)	PA session 1			

Week 2			Training 1	Training 2	

Week 3			Training 3	Training 4	Sports session 1

Week 4			Training 5	Training 6	

Week 5		PA session 2	Training 7	Training 8	

Week 6			Training 9	Training 10	

Week 7			Training 11	Training 12	

Week 8			Training 13	Training 14	

Week 9			Training 15	Training 16	

Week 10		PA session 3	Training 17	Training 18	Sports session 2

Week 11			Training 19	Training 20	

Week 12			Training 21	Training 22	

Week 13			Training 23	Training 24	

Week 14	Post-test 1 (T3)				

Week 15		PA session 4			

Week 16					

Week 17					(Sports session 3)

Week 18					

Week 19					

Week 20		PA session 5			

Week 21					

Week 22					

Week 23					

Week 24					(Sports session 4)

Week 25		PA session 6			

Week 26	Post-test 2 (T6)				

Week 52	Follow-up test (T12)				

Note: After 13 weeks the possibility of continuing fitness training in the day-tot-day situation will be explored for each participant

### Primary outcome measures

#### Level of daily physical activity

**Figure 2:**
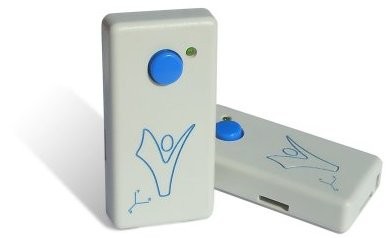
**VitaMove recorder**.

To objectively measure the level of daily PA the VitaMove (VM) system (2 M Engineering, Veldhoven, The Netherlands) will be applied. This system is based on long-term ambulatory monitoring of signals from body-fixed accelerometers. This system consists of 3 to 5 recorders (Figure 2), each with its own accelerometer (Freescale MMA7260Q, Denver, USA), storage capacity and power supply. The recorders are wirelessly connected and synchronized every 10 seconds. Accelerometer signals will be stored digitally with a 128 Hz sampling frequency on a micro Secure Digital (SD) memory card with two gigabytes of storage. Measurements will be uploaded to a computer for kinematic analysis using VitaScore Software (Vitascore BV, Gemert, The Netherlands). A detailed description of the activity detection procedure has been described elsewhere [34]. From the accelerometer signals, the duration, rate, and moment of occurrence of stationary activities (e.g. lying, sitting and standing), dynamic activities (e.g. walking [including climbing/descending stairs], running, cycling, manual wheelchair-driving [including hand-cycling], and general non-cyclic movement) and transitions between postures can be automatically and separately detected with a 1-second resolution. Furthermore, from each measured signal, information on the variability of the acceleration signal (motility, which is related to the intensity of body-segment movements) will be obtained. The VM system is valid to quantify mobility-associated activities and to detect inter-group differences in levels of daily PA [34,35].

For ambulatory participants 3 recorders will be used. One recorder will be attached to each thigh to detect acceleration in anterior-posterior direction while standing and a third recorder will be attached to the sternum to detect acceleration in the anterior-posterior direction and in the longitudinal direction. For participants who use wheelchairs, one additional recorder will be attached to each wrist to detect acceleration in the longitudinal direction while seated with the forearm horizontal in the mid-pronation/supination position. Participants will wear the VM system for 72 hours on randomly selected weekdays while performing their ordinary activities with the exception of swimming and bathing. In addition, the VM system will not be worn during periods of sleep. The recorders will be attached to the body by using elastic belts. The VM system will be set up at each participant's home to minimize influencing the normal PA pattern. To avoid measurement bias, we will instruct the participants to continue their ordinary daily life activities. The principles of the activity monitor will be explained to the participants after all measurements have been made.

The following data will be analyzed from these measurements: (*1*) duration of dynamic activities as a percentage of a 24-hour period; (*2*) number of transitions (includes all transitions except lying transitions between the prone and supine positions); (*3*) intensity of activities: (*3*a) mean motility (in gravitational acceleration (g)); (*3*b) motility during walking; (*3*c) motility during wheelchair-driving; and (*4*) distribution of continuous dynamic activity periods (5-10 sec; 10-30 sec; 30-60 sec; 1-2 min; 2-5 min; 5-10 min; or > 10 min).

#### Self-reported level of physical activity

To measure the self-reported level of PA the Physical Activity Scale for Individuals with Physical Disabilities (PASIPD) [36] will be administered. The PASIPD is a 7-day recall questionnaire developed for people with a physical disability and consists of questions on leisure time, and household- and work-related PA. The questionnaire is translated into Dutch and question 10 (lawn work or yard care) and 11 (outdoor gardening) are combined into a single question, which better represents the Dutch situation. The total score of the PASIPD is created by multiplying the average hours per day for each item by a metabolic equivalent (MET) value associated with the intensity of the activity. The test-retest reliability of the PASIPD is good and its validity is comparable to well-established self-report PA questionnaires for the general population [37].

#### Physical fitness

#### A) Physical fitness: Aerobic capacity

The aerobic capacity will be measured during a maximal ramp protocol. This test will be performed on an electronically braked cycle ergometer or electronically braked armcrank ergometer depending on the main mode of ambulation during daily life, as this elicits the highest oxygen uptake [38]. The test will be preceded by a 3-minute warm-up without resistance. The resistance will be increased every 12 seconds during the test. The magnitude of this increase in resistance depends on the GMFCS level and gender of the participant and ranges from 1 to 6 watts, ensuring that total exercise time will range from 8 to 12 minutes. The target pedal rate during the test is 70 rpm. Strong verbal encouragement will be given throughout the test. The test will be terminated when the participant voluntarily stops due to exhaustion, or when the participant is unable to maintain the initial pedal/crank rate. Gas exchange and heart rate (HR) will be measured continuously using a breath-by-breath analyzing system, which will be calibrated prior to each measurement. Aerobic capacity is defined as the highest mean oxygen uptake during 30 seconds of exercise (VO_2peak _in ml·min^-1 ^and ml·kg^-1^·min^-1^). Subjective strain will be measured immediately after the final stage by the Borg Scale for Rating of Perceived Exertion [39]. Determining a participant's contra-indications for PA will be assessed prior to the test by use of the Physical Activity Readiness Questionnaire [40].

#### B) Physical fitness: sub-maximal aerobic capacity

The 6 minute walking test [41] will be employed as a measure of sub-maximal aerobic capacity. Patients will be instructed to walk, not run, as far as they can along a 30-meter marked track during a 6-minute period. Non-ambulant participants will perform the test in their wheelchair. Patients are allowed to stop and rest during the test, but will be instructed to resume walking/wheelchair driving as soon as they feel able to do so. The average HR during the test and the covered distance will be registered.

#### C) Physical fitness: Muscle strength

Muscle strength will be measured with a hand-held dynamometer (MicroFet, Hoggan Health Industries Inc, West Jordan, UT, USA) using the "break" testing method. The strength of the hip flexors, hip abductors and knee extensors will be measured in individuals whose main mode of ambulation is walking. The strength of shoulder abductors and elbow extensors will be measured in non-ambulant individuals. The applicator of the dynamometer is held against the distal part of the limb segment, and participants will be asked to build up their maximum force against it. When maximum is reached the examiner applies sufficient resistance to overcome the force exerted by the participant. Both the left and right side will be measured. The lever arm from the joint to the dynamometer will be kept constant by marking the position of the dynamometer on each participant's leg. Each trial lasts approximately 4 seconds, and three repetitions will be performed with 1 minute of rest in between. The average value of the three repetitions will be analyzed.

#### D) Physical fitness: Body composition

Height will be measured barefoot in a standing position. In case of joint contractures, measurements will be performed from joint to joint in a lying position. Body mass of ambulatory participants will be obtained while standing barefoot on a scale and of non-ambulatory participants while sitting on an electronic scale. Body mass index (BMI, kg·m^-2^) will be calculated from height and body mass. Waist circumference (cm) will be measured mid-way between the lowest rib and the iliac crest while standing. Waist circumference will be measured in a sitting position in persons using a wheelchair. Thickness of four skin folds (biceps, triceps, subscapular and suprailiac) will be measured twice on the left side of the body with a Harpenden calliper (Burgess Hill, UK). The mean of the two measurements will be used as representative. Percentage body fat will be predicted from skin fold thickness according to the method of Durnin and Womersley [42]. Non-fasting blood samples of approximately 10 ml will be drawn from the vena antecubiti with a Vacutainer needle and collected into an evacuated serum separator tune II (SST II tube) while the participants are seated. Total serum cholesterol, high density lipoprotein cholesterol and low density lipoprotein cholesterol will be determined from this blood sample.

### Secondary outcome measures

The secondary outcome measures will be assessed for descriptive reasons and to get insight into the working mechanisms of the ALSP intervention. These secondary outcome measures will not be fully described, but will only be mentioned here; spasticity (Ashworth Scale [43]), selectivity [44], fatigue (Fatigue Severity Scale [45], Checklist Individual Strength [46], Visual Analog Scale [47]), manual ability (MACS [48]), sports activities, social participation (Life-H 3.0 [49]), health related quality of life (Short-Form-36 [50]), age, gender, level of education, attitude towards PA, general self efficacy (Generalized Self Efficacy scale [51]), intrinsic motivation (Intrinsic Motivation Inventory [52]), and social support for exercise behaviour (Social support for exercise behaviour Scale [53]).

### Statistics

To evaluate the change in PA level and physical fitness, as well as the secondary outcome measures, multilevel regression analyses will be applied, because these analyses allow for missing values. Another advantage of these analyses is that patient data can be clustered within the participating centers. For all multilevel analyses, MLwiN (Version 1.1, Institute of Education, London, UK) software will be used.

## Discussion

This paper outlines the design, methodology and intervention of a multicenter randomized controlled trial that examines the effectiveness of an intervention that aims to achieve a permanent increase in PA level and improve the fitness level of adolescents and young adults with CP by promoting a behavioral change toward a more active lifestyle. The results of this trial are expected to be presented in 2012.

## Competing interests

The authors declare that they have no competing interests.

## Authors' contributions

The work presented here was a collaboration between all the authors. JS and RB drafted the manuscript. JS, RB and MR have made substantial contributions to the conceptualization and the design of the study and defined the research theme. JM, WS, HR, EL and HS have made valuable contributions to the conceptualization and the design of the study and contributed to the manuscript by revising it critically for important intellectual content. All authors have seen and approved the final manuscript.

## Pre-publication history

The pre-publication history for this paper can be accessed here:

http://www.biomedcentral.com/1471-2431/10/79/prepub
